# Tartaric acid pathways in *Vitis vinifera* L. (cv. Ugni blanc): a comparative study of two vintages with contrasted climatic conditions

**DOI:** 10.1186/s12870-016-0833-1

**Published:** 2016-06-28

**Authors:** Céline Cholet, Stéphane Claverol, Olivier Claisse, Amélie Rabot, Audrey Osowsky, Vincent Dumot, Gerald Ferrari, Laurence Gény

**Affiliations:** Institut des Sciences de la Vigne et du Vin, Université de Bordeaux, EA 4577 Unité de recherche œnologie, France; INRA, ISVV, USC INRA 1366 Œnologie, 210 Chemin de Leysotte, CS 50008, F-33882 Villenave d’Ornon, France; Centre Génomique Fonctionnelle, Université de Bordeaux, Plateforme Protéome, France; Bureau National Interprofessionnel du Cognac, Station Viticole, France

**Keywords:** Ascorbate, L-idonate dehydrogenase, Tartrate, *Vitis vinifera* L, Vintage effect

## Abstract

**Background:**

The acid component of grape berries, originating in the metabolism of malate and tartrate, the latter being less well-known than the former, is a key factor at play in the microbiological stability of wines destined for distillation. Grape acidity is increasingly affected by climate changes. The ability to compare two vintages with contrasted climatic conditions may contribute to a global understanding of the regulation of acid metabolism and the future consequences for berry composition.

**Results:**

The results of the analyses (molecular, protein, enzymatic) of tartrate biosynthesis pathways were compared with the developmental accumulation of tartrate in Ugni blanc grape berries, from floral bud to maturity. The existence of two distinct steps during this pathway was confirmed: one prior to ascorbate, with phases of *VvGME, VvVTC2, VvVTC4, VvL-GalDH, VvGLDH* gene expression and abundant protein, different for each vintage; the other downstream of ascorbate, leading to the synthesis of tartrate with maximum *VvL-IdnDH* genetic and protein expression towards the beginning of the growth process, and in correlation with enzyme activity regardless of the vintage.

**Conclusions:**

Overall results suggest that the two steps of this pathway do not appear to be regulated in the same way and could both be activated very early on during berry development.

**Electronic supplementary material:**

The online version of this article (doi:10.1186/s12870-016-0833-1) contains supplementary material, which is available to authorized users.

## Background

Understanding grape maturity is one of the most important keys for the characterization of a vintage. This understanding allows the determination of the most favorable time to harvest, depending on the desired objective in terms of organoleptic quality. Among the parameters which characterize technological maturity, titratable acidity, due principally to two organic acids, malic and tartaric [1], plays an important role. Whereas the primary metabolism of the grape berry and the malic acid pathway relating to grape maturity have been studied in depth [2–5], very few studies relate to the metabolism of tartaric acid [6–9], and especially to the control of this metabolism under biotic or abiotic stress [10]. In some higher plants, including *Vitis vinifera* L. [11], the tartaric acid pathway is the result of L-ascorbic acid (vitamin C) catabolism [8, 12] via the conversion of L-idonate to 5-keto-D-gluconate [13] under the action of L-idonate dehydrogenase (L-IdnDH), the only enzyme of this pathway known at the present time [8]. In *Vitis vinifera* L.*,* the synthesis of tartaric acid occurs in the early stages of grape berry development [3], tartaric acid being found in very small quantities in berries at these early stages [14]. Grape berries accumulate tartaric acid in their pulp [14], which strongly impacts the taste and organoleptic qualities of the resulting juice and the final product [1].

Recent studies on climate change in the French region of Cognac have brought to light a significant increase in temperatures over recent years [15]. Many studies carried out on different grape varieties, have demonstrated that one of the main consequences of abiotic stress in grapes resulting from this change is a decrease in acidity [16, 17], rendering wine preservation and storage more problematic [18, 19]. This is especially true for wine destined for distillation, as in the case of Ugni Blanc wine for Cognac (Charente county, France), as these wines must (under French law) be stored in tanks without sulfites prior to distillation (decree n°2015-10 of 07 January 2015), which means they must have a high level of acidity in order to avoid microbiological spoilage or the development of olfactory defects following distillation.

**Fig. 1:**
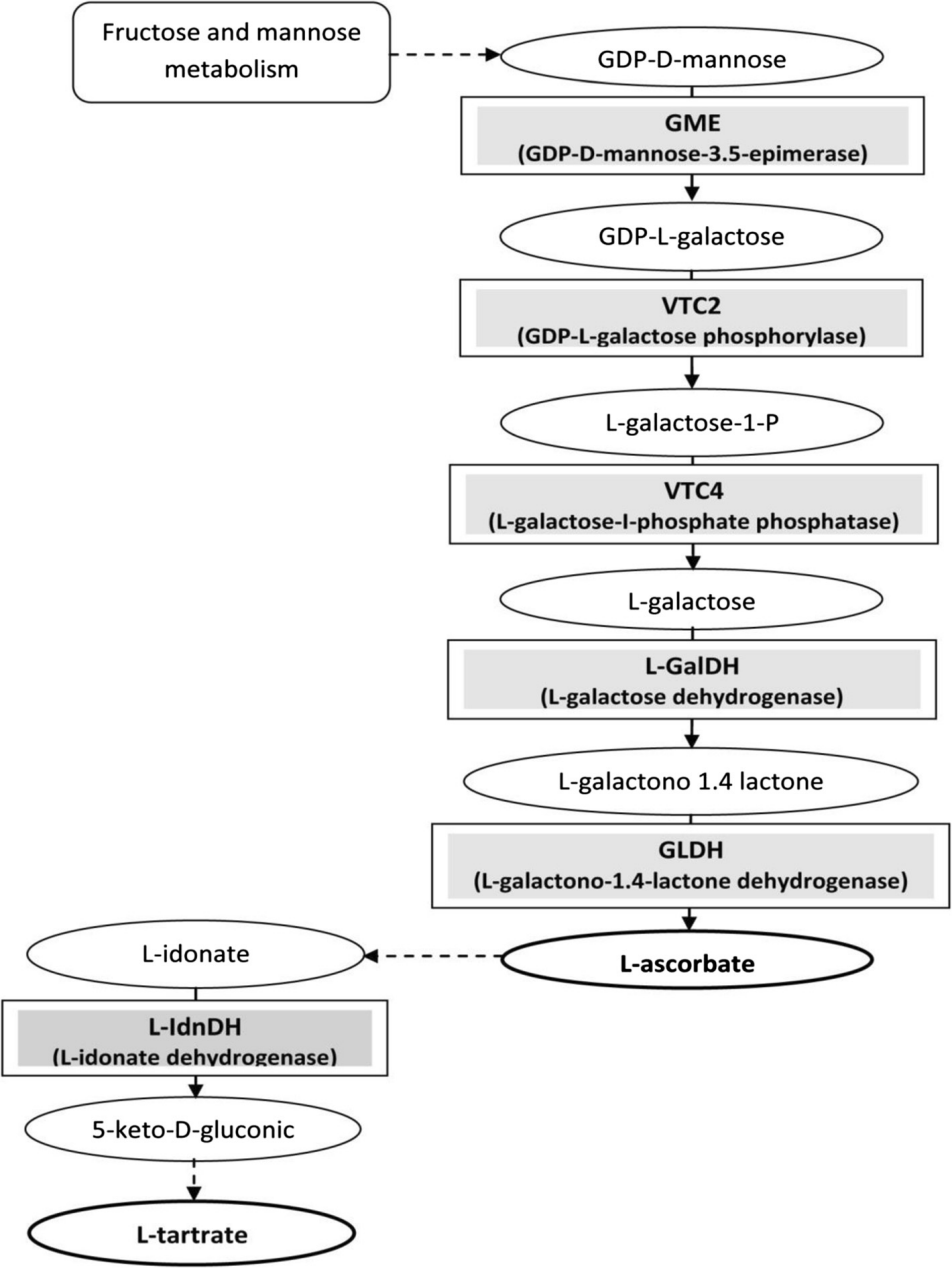
Biosynthesis pathway of L-tartrate, via L-ascorbate (from [8, 9, 21])

While few studies have examined tartaric acid, more and more are focusing on its precursor, ascorbic acid, a multifunctional metabolite essential for growth and development, and also a vital antioxidant involved in defense of the plant against abiotic stress [20–23]. It is now known that the principal pathway for ascorbate synthesis is the result of photosynthesis-based carbon flux via a series of enzyme catalyzed reactions known as the Smirnoff-Wheeler pathway [24, 25]. This pathway, which is dominant during the growth phase of grape berries [9, 21, 26], involves five enzymes which are more or less well known (Fig. 1), and whose locations and key roles still raise many questions. The GDP-D-mannose 3,5epimerase (GME), which is the first enzyme of the pathway, appears to be a key enzyme in the regulation of the biosynthetic pathway of ascorbate [27, 28] since it may control the carbon flux directed towards the synthesis of ascorbate as a function of the redox state of the cell and stress conditions [29]. GME also appears to be a key enzyme in the biosynthetic pathway of cell-wall compounds. It is a “node” between the metabolism of ascorbic acid and of the cell-walls since it may control the availability of sugar for the biosynthesis of cell-wall non-cellulosic polysaccharides [28, 30]. GDP-L-galactose phosphorylase (VTC2), the last enzyme in the Smirnoff-Wheeler pathway to be identified [31, 32], appears to have a partially nuclear localization, suggesting that it may also be a key regulator of this pathway at the nucleus level. The role of L-galactose-1-phosphate dehydrogenase, VTC4, remains unclear in the regulation of ascorbate synthesis [33–35]. Similarly, L-galactose dehydrogenase (L-GalDH) appears to have, in vivo, no more than a moderate influence on the flux of the ascorbate synthesis pathway [34, 36]. Finally, L-galactono-1,4-lactone dehydrogenase (GLDH) intervenes in the last stages of L-ascorbate biosynthesis. This enzyme is specific to the catalysis of L-galactono-1,4-lactone into L-ascorbate [25, 37] from the outer side of the mitochondrion inner-membrane (as reviewed in [21]). GLDH uses the cytochrome *c* in the respiratory chain as an electron acceptor, so it may play an important role in growth regulation [38] and in the regulation of ascorbate synthesis by light [10, 39, 40]. There has been much work on the expression of genes in the pathway of L-ascorbate biosynthesis in plants [34, 35], including in grapes. In contrast, very little work to date has focused specifically on the tartaric acid synthesis pathway in grapes [41, 42]. In *Vitis vinifera* L., the gene expression of *VvL-IdnDH* has already been studied by Deluc et al. [43], and coupled to changes in the translation level of this gene [5, 41]. More recently, Jia et al. [44] highlighted the close similarity between three isoforms of L-IdnDH in grapevine. *In silico* analysis demonstrated that two isoforms are more or less similar (respectively 99 and 77 %) to the L-IdnDH identified by Debolt et al. [8]. Very little research has focused on the quantitative protein of ascorbic and/or tartaric acid pathways in the grapevine. To date, only Martìnez-Esteso et al. [5] have approached the evolution of protein expression from *VvGME* and *VvL-IdnDH* during grape berry development. No research to date has studied the link between transcription levels, translation levels, and enzyme activity levels.

The aim of this present work is to consider the biosynthetic pathway of tartaric acid, via the Smirnoff-Wheeler pathway which is dominant when the synthesis of tartaric acid takes place. The lack of information on links between transcription levels, translation levels, and enzyme activity levels led to the initiation of this study with a view to improving knowledge of how this pathway functions. Two contrasted vintages were studied by combining, on the one hand, the study of the relative expression and translation levels of the six genes involved in this pathway and, on the other hand, the changing activity levels of the L-IdnDH enzyme as well as ascorbic and tartaric acid content in Ugni blanc grape berries. The mechanism of regulation will be discussed in the context of global warming, the aim being to prevent too great a drop in the total acidity of musts and, subsequently, of wines.

## Results

### Climate conditions of vintages and physiological parameters of berries

**Fig. 2:**
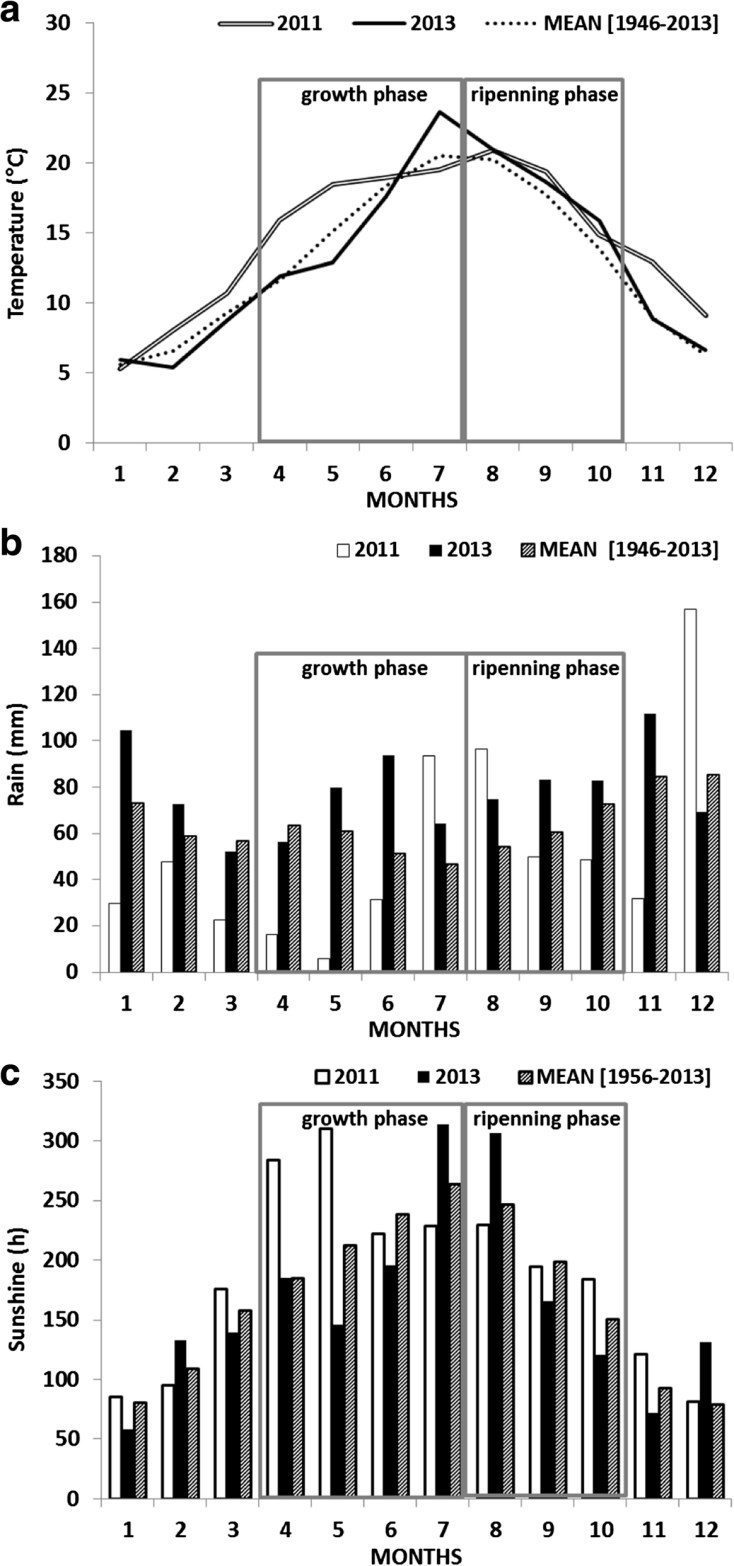
Climatic parameters during the two vintages studied, compared with the last fifty years mean. **a**: temperature (°C: degrees Celsius); **b**: rain (mm: millimeters); **c**: sunlight (h: hours)

In comparison with the fifty last vintages, the 2011 vintage was early, hot and dry. The herbaceous-growth period was very sunny (Fig. 2c, 1045 h) with low rainfall (Fig. 2b, 147 mm) and temperatures were high (Fig. 2a) for the season. The maturation period was hot (Fig. 2a) and humid with higher-than-average rainfall at the beginning of this phase (Fig. 2b, 195 mm; 607 h). On the other hand, 2013 was a later vintage, the herbaceous-growth period being wet (Fig. 2b, 295 mm), rather cool (Fig. 2a) and with little sun (Fig. 2c, 841 h). The maturation period was hot (Fig. 2a) with relatively high rainfall (Fig. 2b, 241 mm). These contrasted conditions contributed to the “vintage effect” corresponding to the interaction between the plant, the soil and climate components. Vintage effect explains the differences between wines produced in different years.

During the herbaceous-growth phase, berry weights were lower in 2011 as compared to 2013 (Table 1, 2011/2013 respectively: 204/359 mg on average), reflecting the water constraint of young berries during their early growth [45, 46]. During the maturation phase, this trend was reversed from the end of veraison up to the harvest due to the rainfall in August. Berry total acidity was lower in 2011 than in 2013 (Table 2) due possibly to the water constraint during the growth phase.

**Table 1:** Developmental profile of berry weight during grape berry development

Berry weight (mg)	FB	PS	BC	VB	VE	H
Vintage 2011	7 ± 1	111 ± 3	495 ± 2	844 ± 112	1254 ± 143	2000 ± 197
Vintage 2013	8 ± 1	129 ± 3	940 ± 81	1233 ± 31	1510 ± 32	1822 ± 73

Unit = milligram (mg). Values are the mean (±SD) of 20 whole berries randomly picked and weighed for each development stage; *FB* flowering beginning, *PS* pea-sized, *BC* bunch closure (berries touching), *VB* veraison beginning (10 % ripe berries), *VE* veraison end (80 % ripe berries), *H* harvest (maturity)

**Table 2:** Developmental profile of total acidity during the maturation phase

Total acidity (g. L-1 of H2SO4)	VB			VE			H	
Vintage 2011	59DAA	66DAA	73DAA	81DAA	87DAA	94DAA	101DAA	108DAA
Vintage 2013	25.1	20.3	15.7	11.4	8.6	7.2	6.1	6.2

Unit = gram of H_2_SO_4_ per liter of must (g. L^−1^ of H_2_SO_4_). Values are the mean of 20 grapes randomly collected at regular intervals from the beginning of veraison until the harvest, pressed, and the musts analyzed; *DAA* days after anthesis

### Developmental accumulation of total ascorbic and tartaric acids

**Fig. 3:**
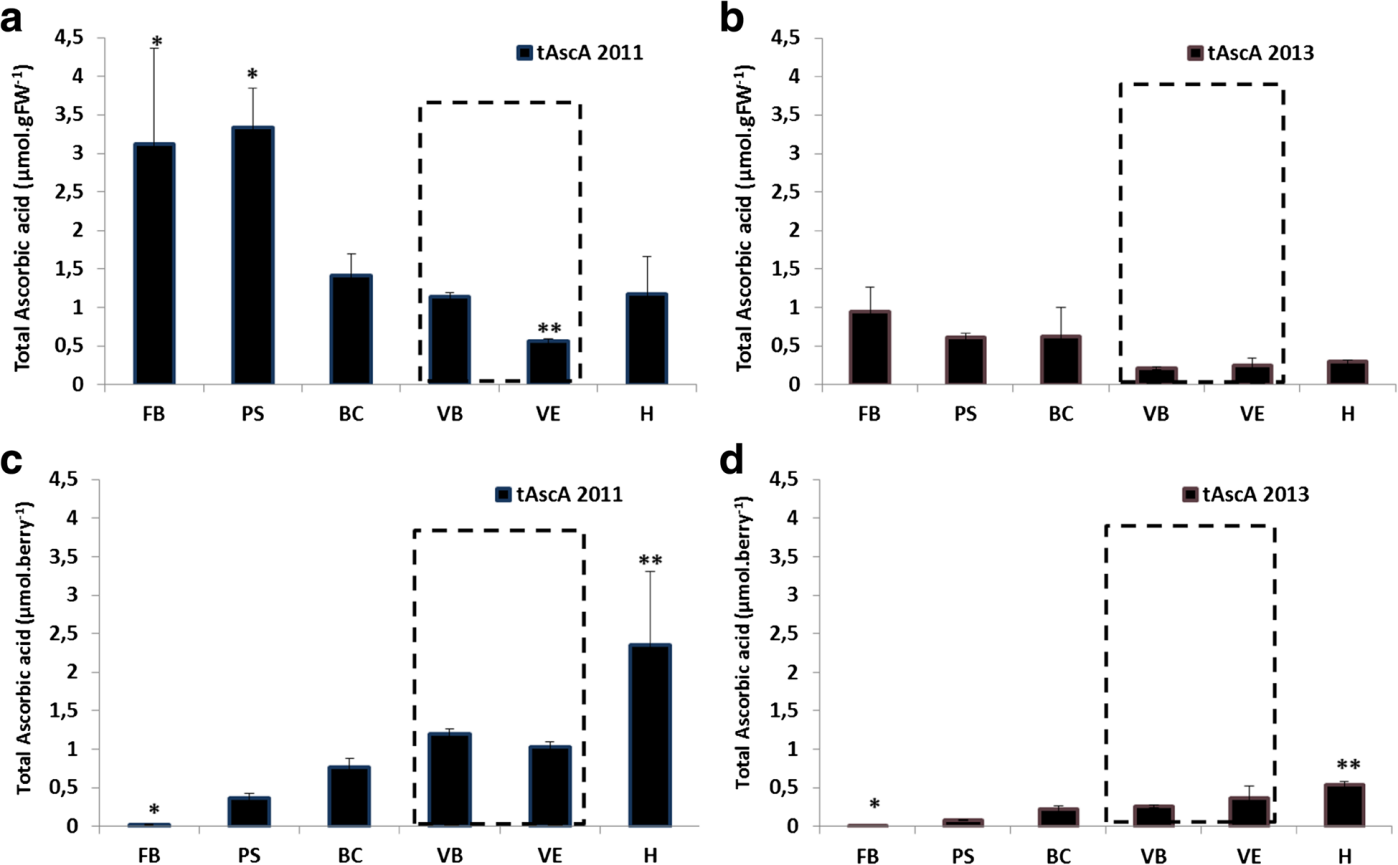
Total ascorbic acid content during grape berry development for the two vintages of the study. Error bars are standard errors of three biological replicates and three technical (HPLC analysis) replicates. The developmental stage of veraison is indicated by a grey dotted box. (*: significant difference in Friedman test (α = 0.05)). **a**: data 2011 expressed in micromoles per gram of fresh weight (μmol.gFW-1); **b**: data 2013 expressed in micromoles per gram of fresh weight (μmol.gFW-1); **c**: data 2011 expressed in micromoles per berry (μmol.berry-1); **d**: data 2013 expressed in micromoles per berry (μmol.berry-1). FB: flowering beginning; PS: pea-sized; BC: bunch closure (berries touching); VB: veraison beginning (10 % ripe berries); VE: verasion end (80 % ripe berries); H: harvest (maturity)

Ascorbic acid (Fig. 3) is the only known precursor of tartaric acid. In 2011 (Fig. 3a, c), an early accumulation of ascorbic acid in the berry was reflected by a first phase with high concentration of ascorbic acid. This phase covered the beginning of the herbaceous growth period of the grape berry (FB: 3.1 ± 1.2 μmol.gFW^−1^; PS: 3.3 ± 0.5 μmol.gFW^−1^). A second phase, with a marked drop in ascorbic acid levels, was observed at the bunch closure stage until the end of veraison (from 1.4 ± 0.3 μmol.gFW^−1^ for BC, to 0.6 ± 0.1 μmol.gFW^−1^ for VE), which is in accordance with the multiple roles of this acid in plant metabolism [47]. During maturation, a third phase showing a slight increase in concentrations was brought to light (H: 1.2 ± 0.5 μmol.gFW^−1^). In 2013 (Fig. 3b, d), level contents tended to have a similar profile to those of 2011, but only for the two first phases. The first phase covered the entire period of herbaceous growth of the grape berry. The second phase was initiated at the start of veraison, with levels of ascorbic acid remaining unchanged until the harvest (VB/VE/H respectively (μmol.gFW^−1^): 0.21 ± 0.02; 0.25 ± 0.09; 0.29 ± 0.03). In both the 2011 and 2013 vintages, profiles of ascorbic acid content per berry (μmol.berry^−1^) increased significantly from flowering until maturity (126-fold in 2011: from 0.02 ± 0.01 for FB, to 2.35 ± 0.03 for H; 6.7-fold in 2013: from 0.08 ± 0.01 for FB, to 0.53 ± 0.05 for H), in accordance with the evolution of berry growth (Table 1).

**Fig. 4:**
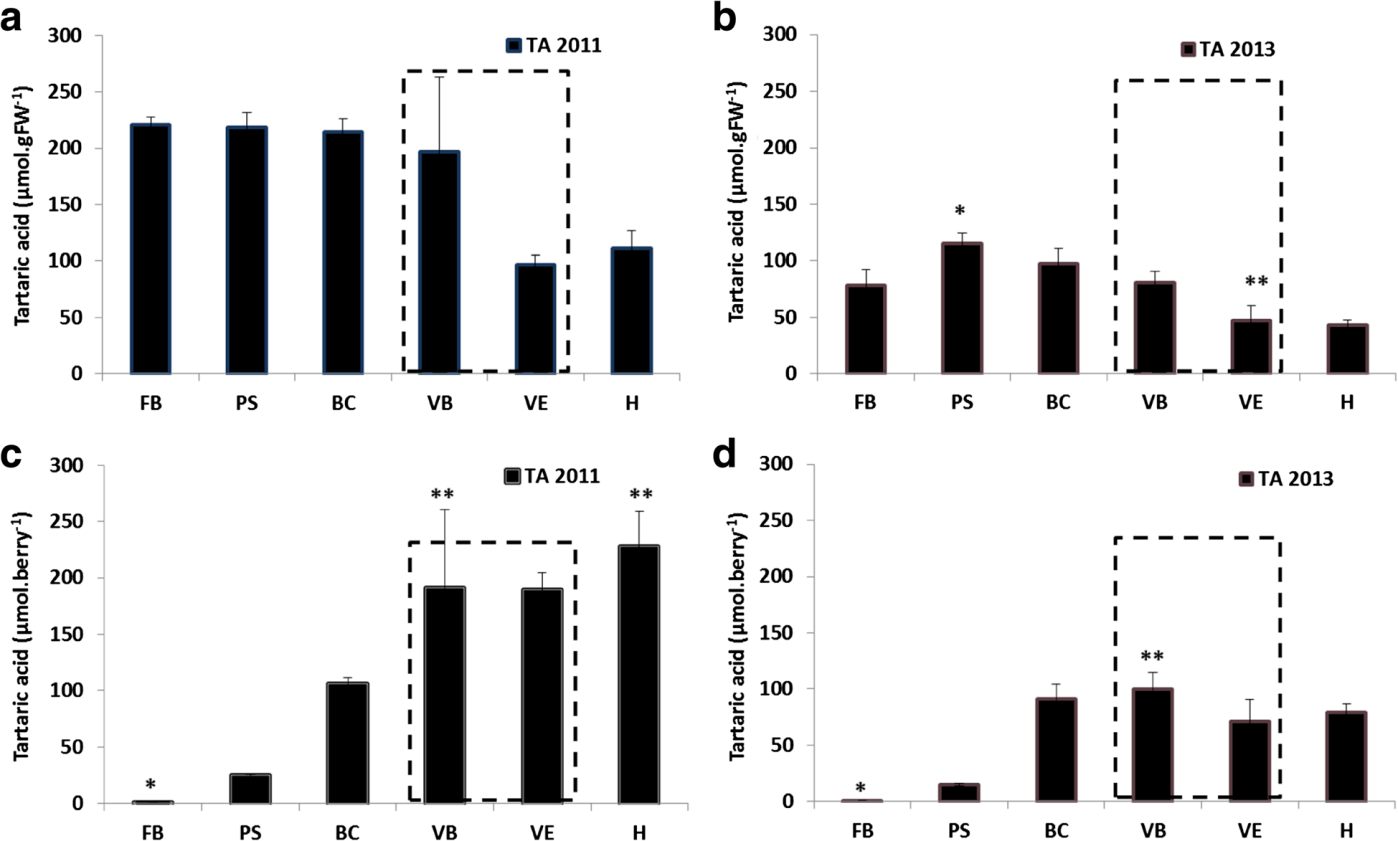
Tartaric acid content during grape berry development for the two vintages of the study. Error bars are standard errors of three biological replicates and three technical (HPLC analysis) replicates. The developmental stage of veraison is indicated by a grey dotted box. (*: significant difference in Friedman test (α = 0.05)). **a**: data 2011 expressed in micromoles per gram of fresh weight (μmol.gFW-1); **b**: data 2013 expressed micromoles per gram of fresh weight (μmol.gFW-1); **c**: data 2011 expressed in micromoles per berry (μmol.berry-1); **d**: data 2013 expressed in micromoles per berry (μmol.berry-1). FB: flowering beginning; PS: pea-sized; BC: bunch closure (berries touching); VB: veraison beginning (10 % ripe berries); VE: verasion end (80 % ripe berries); H: harvest (maturity)

The correlation between ascorbic acid accumulation (Fig. 3) and tartaric acid profiles (Fig. 4) was evident (μmol.gFW^−1^ and μmol.berry^−1^). Similarly to ascorbic acid content, an accumulation of tartaric acid (μmol.gFW^−1^) occurred at an early stage in the growth phase. In 2011 (Fig. 4a, c), an increase in the concentration of tartaric acid occurred up to the beginning of veraison (FB: 220.6 ± 7.0 μmol.berry^−1^; PS: 218.3 ± 12.9 μmol.berry^−1^; BC: 214.6 ± 11.0 μmol.berry^−1^; VB: 196.7 ± 66.1 μmol.berry^−1^), then the concentration tended to decrease until the end of veraison (VE: 97.0 ± 8.2 μmol.berry^−^1), after which it remained unchanged until harvest (H: 110.9 ± 15.9 μmol.berry^−1^). The profile of tartaric acid quantity per berry increased significantly from flowering until the beginning of veraison (from 1.5 ± 0.1 μmol.berry^−1^ for FB, to 90.9 ± 7.0 μmol.berry^−1^ to VB), in accordance with berry herbaceous growth (Table 1). During the maturation phase the quantity increased anew up to harvest (H: 227.5 ± 31.8 μmol.berry^−1^). In 2013 (Fig. 4b, d) a peak of concentration was observed at pea-size stage (PS: 115.2 ± 9.0 μmol.berry^−1^), followed by a slight decrease until the end of veraison (VE: 47.4 ± 12.6 μmol.berry^−1^). The profile of quantity per berry increased significantly from flowering until the beginning of veraison (from 0.6 ± 0.1 μmol.berry^−1^ at FB, to 100.2 ± 14.6 μmol.berry^−1^ at VB), as in 2011. However, during the maturation phase, the quantity decreased and then tended to remain unchanged until harvest (VE: 71.5 ± 18.9 μmol.berry^−1^; H: 79.1 ± 7.9 μmol.berry^−1^).

Moreover, quantity of total ascorbate and tartrate, and their concentrations were found to be two or three times greater (depending on the stage of development) in 2011 than in 2013, which could suggest a vintage effect.

### Developmental expression of gene-encoding enzymes of the ascorbic and tartaric acid pathways

**Fig. 5:**
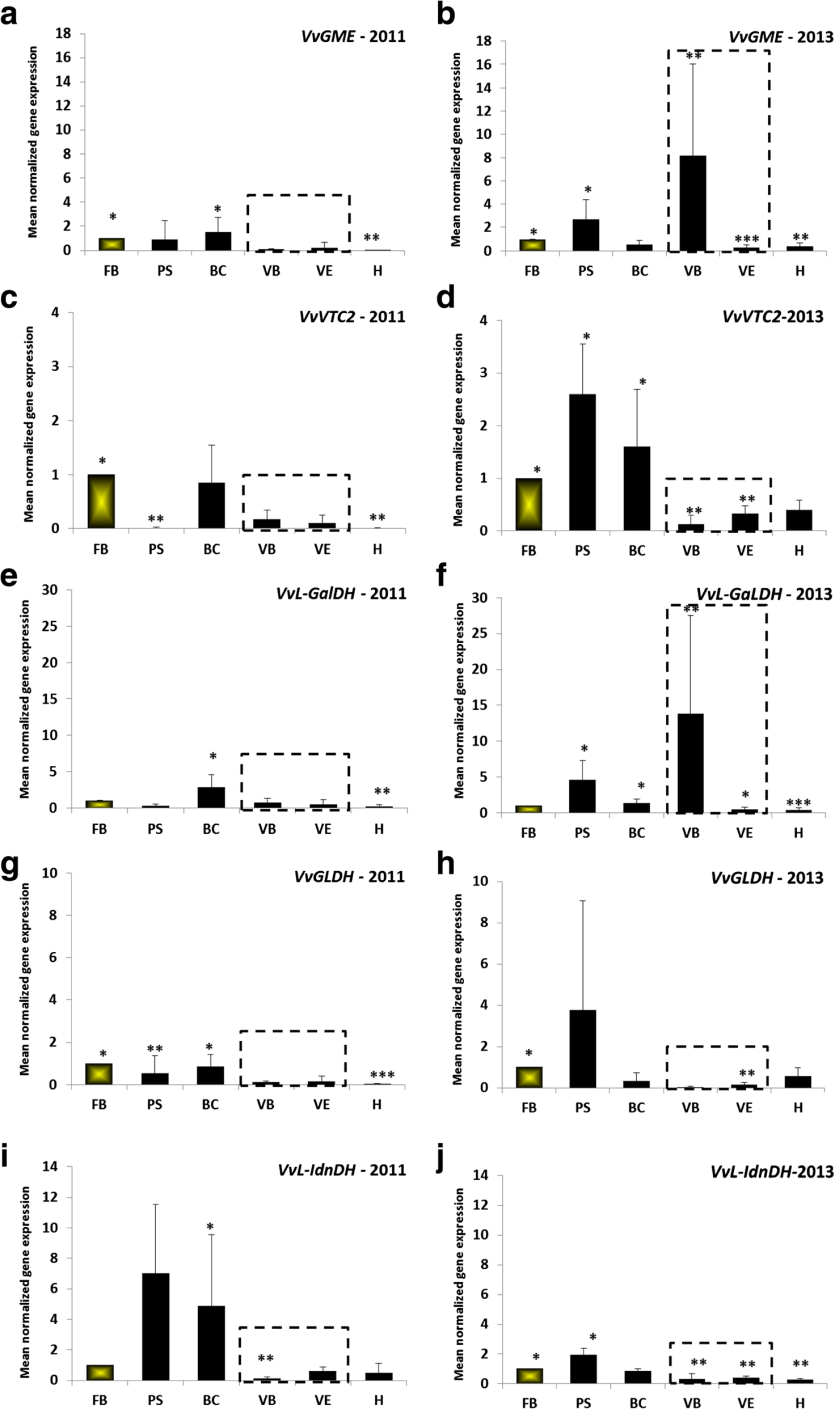
Normalized mean (with UBi and EFi) of transcription of specific genes during grape berry development. GDP-D-mannose-3, 5 epimerase (*VvGME*) during 2011 (**a**) and 2013 (**b**); GDP-L-galactose phosphorylase (VvVTC2) during 2011 (**c**) and 2013 (**d**); L-galactose dehydrogenase (*VvL-GalDH*) during 2011 (**e**) and 2013 (**f**); L-galactono-1,4-lactone dehydrogenase (*VvGLDH*) during 2011 (**g**) and 2013 (**h**); L-idonate dehydrogenase (*VvL-IdnDH*) during 2011 (**i**) and 2013 (**j**). The developmental stage of veraison is indicated by a grey dotted box. The reference stage is indicated by the colored histogram. Error bars are standard errors of three biological replicates and duplica technical (Q-PCR analysis) replicates. (*: significant difference in Friedman test (α = 0.05)). FB: flowering beginning; PS: pea-sized; BC: bunch closure (berries touching); VB: veraison beginning (10 % ripe berries); VE: verasion end (80 % ripe berries); H: harvest (maturity); GME: GDP-D-mannose-3, 5 epimerase; VTC2: GDP-L-galactose phosphorylase; L-GalDH: L-Galactose dehydrogenase; GLDH: L-galactono-1,4-lactone dehydrogenase; L-IdnDH: L-Idonate dehydrogenase

For both the 2011 and 2013 vintages, it was observed that profiles of expression of the gene-encoding enzymes in the Smirnoff-Wheeler pathway (i.e. *VvGME, VvVTC2, VvGLDH, VvL-GalDH)* were comparable (Fig. 5a, b, c, d, f, g, h). They were characterized by a first phase corresponding to an increase in the transcription level of the four genes during herbaceous growth. In 2011 the profiles of these gene expressions rose to a maximum at bunch closure (BC: *VvGME*: 1.5 ± 1.2; *VvVTC2*: 0.9 ± 0.7*, VvGLDH*: 0.9 ± 0.6*, VvL-GalDH*: 2.8 ± 1.7). Following this increase, the expression of the same genes decreased from the start of veraison until the end of the maturation phase (*VvGME*: 75-fold; *VvVTC2*: 141-fold; *VvGLDH*: 43-fold; *VvL-GalDH*: 14-fold). In 2013 expression profiles of the four genes were more intense and less homogenous than those of 2011; during the herbaceous growth phase, one peak of expression occurred at the pea-size stage (PS: *VvGME*: 2.7 ± 1.7; *VvVTC2*: 2.6 ± 0.9*, VvGLDH*: 3.6 ± 5.3*, VvL-GalDH*: 4.6 ± 2.7), and was earlier than in 2011. Following this, during the maturation phase, the *VvGME* and *VvL-GalDH* expression profiles showed a maximum expression at the beginning of veraison (VB: *VvGME*: 8.2 ± 7.8; *VvL-GalDH*: 13.9 ± 13.7) which was not observed in 2011. Therefore this second phase of expression level increase might be the result of a vintage effect.

However, the *VvL-IdnDH* expression profile (Fig. 5i, j) differed from that of the previous 4 genes, for both the 2011 and 2013 vintages. In 2011 a high level of *VvL-IdnDH* expression occurred from the beginning of the growth phase until the bunch closure stage (BC: 4.9 ± 4.7), which was followed by a significant drop in the transcription level of *VvL-IdnDH* up to the beginning of veraison (35-fold lower at VB). In 2013, the level of *VvL-IdnDH* expression during the herbaceous growth phase was less intense than that of 2011, which might demonstrate a vintage effect. A high level of gene expression was found during this phase, as was the case in 2013, but the maximum appeared earlier, at the pea-size stage (PS: 1.9 ± 0.5). Following this peak, the level decreased until the bunch closure stage, thereafter tending to remain unchanged until harvest (from 0.9 ± 0.1 at BC, to 0.3 ± 0.04 at H).

### Developmental translation of ascorbic and tartaric acid gene-encoding enzymes

**Fig. 6:**
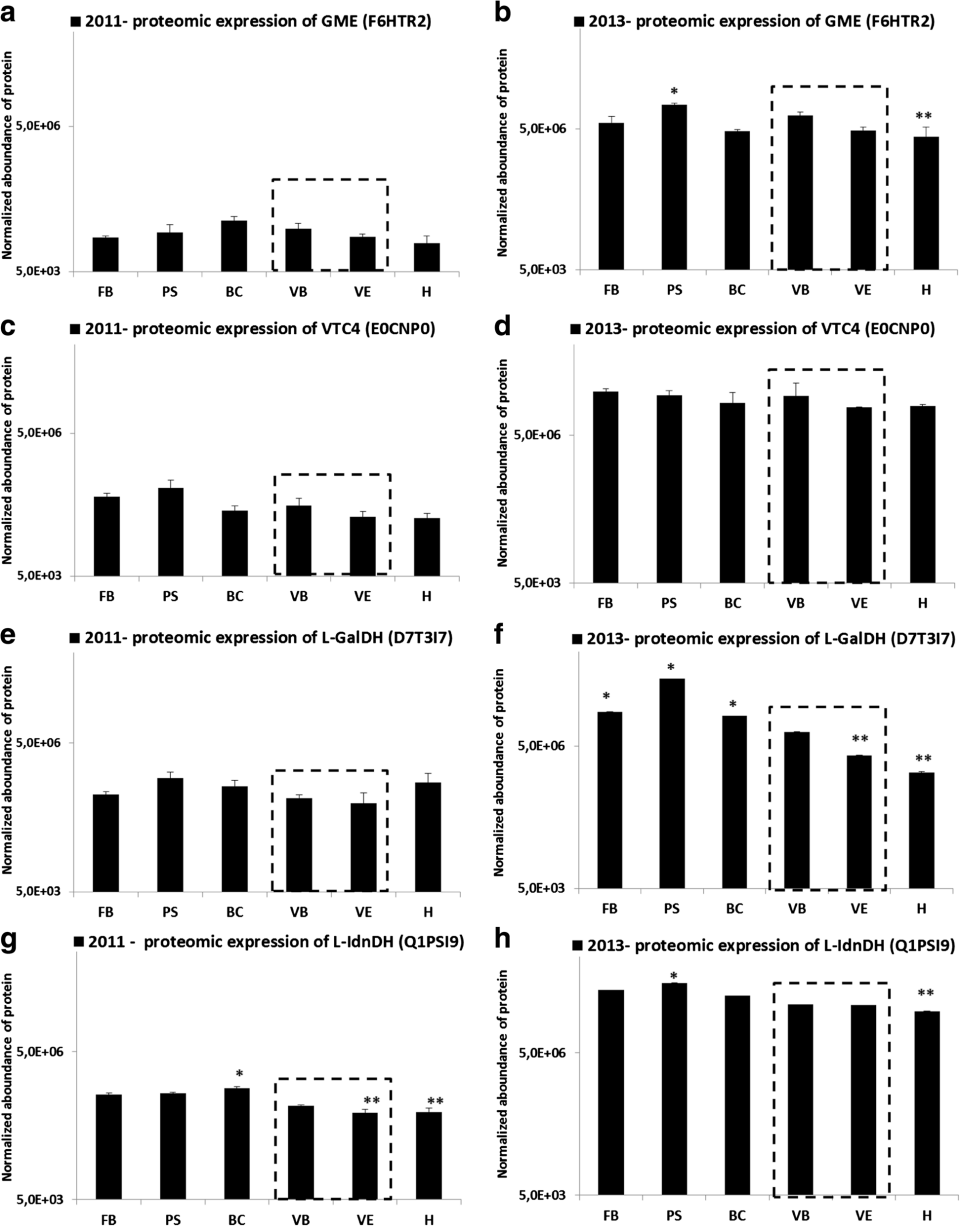
Normalized abundance of specific proteins during grape berry development. GDP-D-mannose-3,5epimerase (GME, uniprot ID:F6THR3) during 2011 (**a**) and 2013 (**b**); L-galactose-1-phosphate phosphatase (VTC4, uniprot ID: E0CNP0) during 2011 (**c**) and 2013 (**d**); L-galactose dehydrogenase (L-GalDH, uniprot ID: D7T3I7) during 2011 (**e**) and 2013 (**f**); L-idonate dehydrogenase (L-IdnDH, uniprot ID: Q1PSI9) during 2011 (**g**) and 2013 (**h**). Error bars are standard errors of three biological replicates (LabelFree analysis). The developmental stage of veraison is indicated by a grey dotted box. (*: significant difference in Friedman test (α = 0.05)). FB: flowering beginning; PS: pea-sized; BC: bunch closure (berries touching); VB: veraison beginning (10 % ripe berries); VE: verasion end (80 % ripe berries); H: harvest (maturity); GME: GDP-D-mannose-3, 5 epimerase; VTC4: L-galactose-1-phosphate dehydrogenase; L-GalDH: L-Galactose dehydrogenase; L-IdnDH: L-Idonate dehydrogenase

The migration of the protein samples was stopped before their separation on the gel, thus no information about the size of their peptides is available. In the present study, MS was quantified with the aim of measuring the partial acid metabolism from proteome, comprising the four major soluble proteins belonging to the biosynthetic pathway of tartaric acid: GME, VTC4, L-GalDH, L-IdnDH. The four proteins of interest were detected at all stages of development in both the 2011 and 2013 vintages, with greater abundance during 2013 which could be the result of a vintage effect (Fig. 6).

Firstly, there was a difference between the abundance profiles of the three enzymes involved in the synthesis of ascorbic acid. In 2011, the GME profile (Fig. 6a) was constant until harvest (around 0.03E^+6^), with a non-significant peak at the bunch closure stage. In contrast, 2013 GME abundance (Fig. 6b) was generally higher (around 7.7 E^+6^) showing a contrasted pattern throughout the season; GME decreased significantly from ea-size until harvest (from 16.6E^+6^ ± 1.3E^+6^ at PS, to 3.4E^+6^ ± 2.1E^+6^ at H). The evolution of VTC4 profiles was similar for the two vintages (2011: Fig. 6c; 2013: Fig. 6d). Nevertheless, both profiles differed from those of GME: the levels of abundance remained constant from the beginning of flowering (FB) until harvest (H) (around 0.02E^+7^ in 2011; 2.7E^+7^ in 2013). Concerning L-GalDH, the profile evolution was constant in 2011 (Fig. 6e) as was the case for GME. For the 2013 vintage (Fig. 6f), a high level of protein abundance was measured (around 3.3E^+7^) during herbaceous growth, with a significant peak at the pea-size stage (PS: 13.2E^+7^ ± 3.4E^+7^). Following that, a drop in L-GalDH abundance from the end of veraison until the maturity of the berry was detected (from 1.0E^+7^ ± 0.5E^+7^ at VE, to 0.14E^+7^ ± 0.01E^+7^ at H).

Secondly, in contrast, the protein abundance profile of L-IdnDH was different from that of the enzymes involved in the synthesis of ascorbic acid, particularly in the 2011 vintage (Fig. 6g). Its abundance was observed to remain stable (around 0.8E^+6^ in 2011, and 110E^+6^ in 2013) from the beginning of growth until the end of this phase, with a peak at the bunch closure stage in 2011 (BC: 0.9E^+6^ ± 0.1E^+6^), or at the pea-size stage in 2013 (PS: 150E^+6^ ± 20E^+6^; Fig. 6h). For both vintages, L-IdnDH abundance subsequently decreased during maturation (around 2.33-fold in 2011 and 2.38-fold in 2013).

### Developmental profile of L-IdnDH enzyme activity

**Fig. 7:**
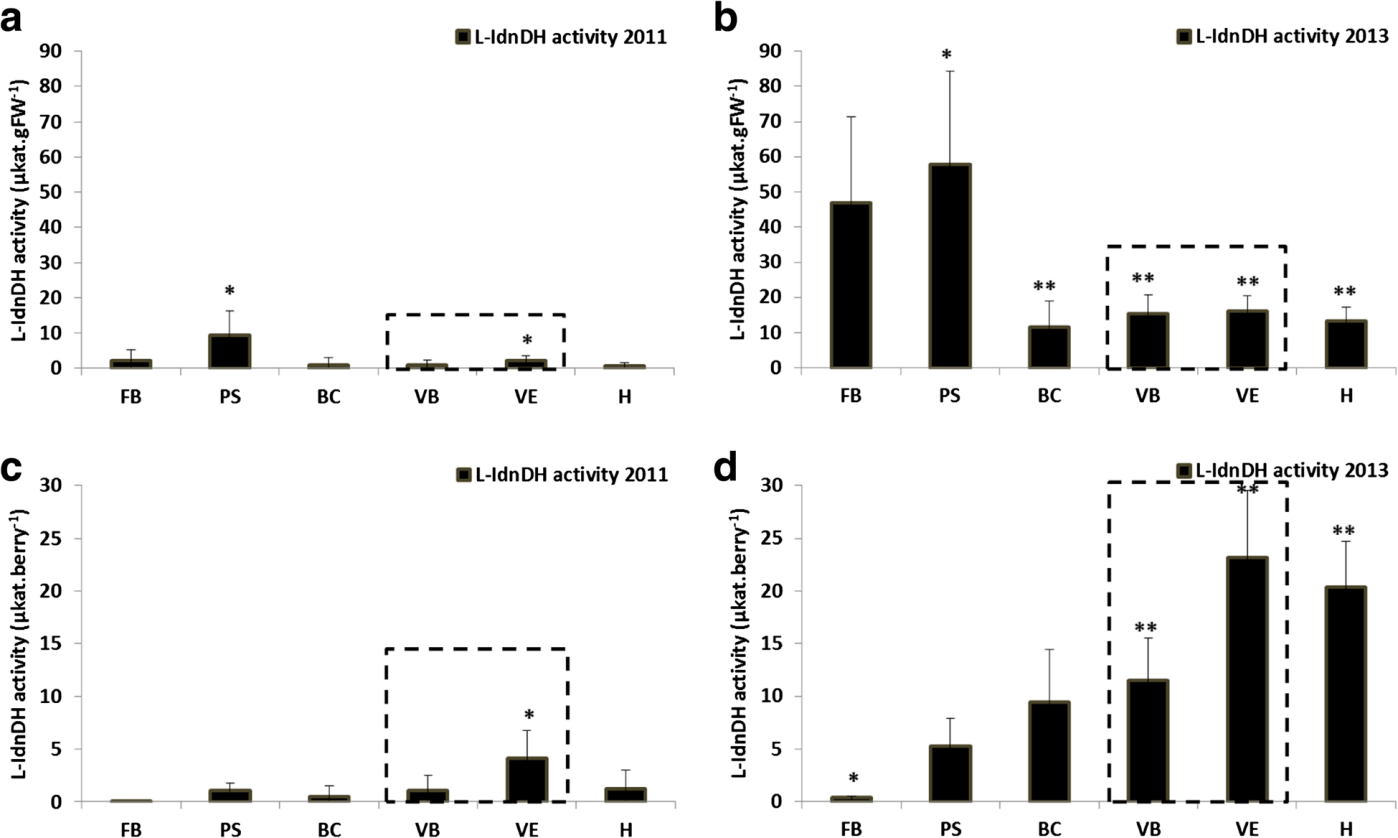
L-IdnDH activity during grape berry development for the two vintages of the study. Error bars are standard errors of three biological replicates and three technical (spectrophotometric analysis) replicates. The developmental stage of veraison is indicated by a grey dotted box. (*: significant difference in Friedman test (α = 0.05)). **a**: data 2011 expressed in microkatal per gram of fresh weight (μkat.gFW-1); **b**: data 2013 expressed in microkatal per gram of fresh weight (μkat.gFW-1); **c**: data 2011 expressed in microkatal per berry (μkat.berry-1); **d**: data 2013 expressed in microkatal per berry (μkat.berry-1). FB: flowering beginning; PS: pea-sized; BC: bunch closure (berries touching); VB: veraison beginning (10 % ripe berries); VE: verasion end (80 % ripe berries); H: harvest (maturity)

In 2011 (Fig. 7a, c), the profile of L-IdnDH enzyme activity showed two peaks separated by a period of very low activity. Globally speaking, the activity occurred at the early stages of vegetative growth, with a maximum at the pea-size stage (PS: 9.5 ± 6.8μkat.gFW^−1^; 1.1 ± 0.8μkat.berry^−1^) followed by a decrease (9.1-fold (μkat.gFW^−1^) and 2-fold (μkat.berry^−1^)) in activity at bunch closure. Curiously, a second peak measured during maturation (VE: 2.3 ± 1.3μkat.gFW^−1^; 4.2 ± 2.6μkat.berry^−1^) may be linked with a vintage effect. In the same way, in the 2013 vintage (Fig.7b, d), the profile of L-IdnDH enzyme activity presented one significant peak during vegetative growth, at pea size (PS: 57.9 ± 26.6μkat.gFW^−1^; 5.3 ± 2.6μkat.berry^−1^), followed by a period of low but stable activity from bunch closure until harvest (from 11.6 ± 7.3μkat.gFW^−1^; 9.5 ± 5.0μkat.berry^−1^ at BC, to 13.4 ± 3.6μkat.gFW^−1^; 20.3 ± 4.4μkat.berry^−1^at H).

The lower level of enzymatic activity in 2011 as compared to 2013 (6.1-fold) indicates the existence of a significant vintage effect.

## Discussion

The overview of all the results assists in the analysis of the interactions between the parameters studied, and some interesting biological observations have been highlighted.

Firstly, in Ugni blanc grape berries, the expression profile of the *VvGME, VvVTC2*, and *VvL-GalDH* genes corresponds to the results obtained with other cultivars [9], but also to those obtained with other plants such apple leaf [35] or tomato fruit [34], which confirms the common phylogenetic heritage of the Smirnoff-Wheeler pathway in higher plants. However, the expression profiles of these genes appeared correlated, as is the case for other fruit varieties [34, 35], but this is not always the case for other *Vitis vinifera* L. cultivars. Indeed, concerning *VvGLDH,* the expression profile was correlated with that of other genes (*VvGME, VvVTC2*, *VvL-GalDH*) in Ugni blanc berries, but in South-Australian Shiraz berries, it was not the case [9]. *VvGLDH* regulation in *Vitis vinifera* L. does not appear to be strongly dependent on the stage of berry development. This regulation could be influenced by environmental effects such as sunlight [10, 39], which is not the same in the Cognac region of France, and in South-Australia. Moreover, this effect could explain the variations in patterns of gene expression observed between 2011 and 2013. Concerning *VvL-IdnDH*, the early peak of expression observed suggests a regulation of this gene in Ugni blanc berries early in their development, prior to flowering, as is the case in other cultivars e.g. Shiraz, Cabernet sauvignon and Chardonnay [9, 10, 41, 42]. This regulation does not seem to be cultivar or environment dependent. On the other hand, *VvL-IdnDH* regulation seems to be connected with the ascorbate level which could play a role of activator of transcriptional regulator. Our results have shown on the one hand that the evolution patterns of the relative expressions of Smirnoff-Wheeler genes do not seem to be correlated with those of *VvL-IdnDH* and, on the other hand, that all Smirnoff-Wheeler genes had higher levels in 2013 as compared to 2011, whereas opposite results were found for *VvL-IdnDH.* These conclusions are confirmed by the vintage effect observed on the levels of relative gene expression. These results suggest that the enzymes that are involved in the tartaric acid synthesis act independently of the enzyme suite involved in the Smirnoff-Wheeler pathway.

Secondly, proteomic analysis of Ugni blanc berries demonstrated that, during the herbaceous growth phase, as is the case for the level of *VvGME* expression, the level of GME abundance was lower in 2011 than in 2013. In the 2011 vintage, the growth phase was drier than in 2013, which explains the smaller berry size [45] in 2011. GME plays a key role in ascorbate biosynthesis but also in the biosynthesis of cell-wall compounds by affecting both cell division and cell expansion [28, 30, 43, 44]. Taken together these data suggest that the degree of involvement of GME in cellular metabolism during the growth phase of grape berries is not the same in different vintages. The ascorbate richness of green berries in 2011 suggests that the functional pathway of GME for the ascorbate biosynthesis takes priority over the biosynthesis of cell-wall compounds, which is coherent with the formation of smaller berries. This metabolic variation may be affected either by water deficit and/or by other parameters linked with the vintage conditions. In addition, as is the case for Smirnoff-Wheeler proteins, the level of L-IdnDH abundance is modified by vintage effect. During the herbaceous growth phase this level was lower in 2011 than in 2013, which is in contradiction with the level of *VvL-IdnDH* expression. It may be that the level of tartrate can have a “feedback” effect on the level of L-IdnDH translation. Thus, a low level of tartaric acid accumulation (as was the case in 2013) could activate L-IdnDH translation, inducing the pathway activation downstream of 5, keto-D-gluconic acid, which may stimulate the production of an unknown inhibitor of tartrate synthesis. In the opposite case, the limitation of the pathway between 5, keto-D-gluconic acid and tartrate would not permit the activation of this inhibitor and would permit tartrate synthesis in large quantities (as was the case in 2011). Put otherwise, protein accumulation patterns are positively correlated for GME and for L-IdnDH (Additional file 1: Figure S1 and Additional file 2: Figure S2), raising the question as to whether GME abundance may induce a regulation of L-IdnDH translation. During the maturation period the quantity of transcripts is similar whatever the genes and the vintages studied while the quantity of proteins is greater in 2013 than in 2011, which suggests the existence of post-transcriptional mechanisms in adaptation to environmental variations. Furthermore, if enzymes appear to be affected by the vintage, the profile of protein abundance seems to be similar for different *Vitis vinifera* L. cultivars such as Chinese Shiraz and Cabernet sauvignon [41] for L-IdnDH, or Spanish Muscat de Hambourg for L-IdnDH and GME [5].

Thirdly, it is interesting to see that, as is the case for Chinese Shiraz and Cabernet sauvignon [41], the first peak of enzyme activity in Ugni blanc berries corresponds to the pea-size stage (PS), suggesting that this activity is probably not dependent on the cultivar. A link was brought to light between the level of protein abundance and intensity of enzyme activity of L-IdnDH, which both seem to depend on a vintage effect. Indeed, protein abundance and enzyme activity were lesser in 2011 than in 2013.

Finally, the profiles of ascorbic and tartaric acid contents of Ugni blanc grape berries were in accordance with those obtained with other cultivars of *Vitis vinifera* L. such as Shiraz, Chardonnay, and Cabernet sauvignon [9, 10, 41, 42]. A strong link was observed in both 2011 and 2013 between tartaric acid and total ascorbic acid, highlighted by the developmental accumulation of these two acids which appeared to be closely correlated. This finding is substantiated by the strong correlation observed in the matrices (Additional file 1: Figure S1, Additional file 2: Figure S2), especially for the 2011 vintage. However, the apparent contradiction between levels of total ascorbate accumulation and abundance of Smirnoff-Wheeler proteins may reflect a “feedback” effect of ascorbate in connection with its other synthesis pathways [25, 47].

### Tartaric acid/ascorbic acid link

The result of the present organic-acid study, supplemented by correlation study, confirmed the existence of a strong link between the metabolisms of tartaric and ascorbic acids as described by DeBolt et al. [8] and Melino et al. [9, 10]. The general shape of the curve of evolution in tartaric-acid levels had the same accumulation phases as that of ascorbic acid and was seemingly dependent on that of its precursor. These profiles were in accordance with those described by Melino et al. [9, 10], reflecting three accumulation phases. A first storage phase covers the entire herbaceous growth of the grape berry. It is at the beginning of the first phase of accumulation (berry herbaceous growth) that the catabolism of ascorbic-acid towards the production of tartaric acid takes place [48], which explains the parallel increase in the levels of ascorbic acid and tartaric acid (expressed in μmol.berry^−1^). During this period the synthesis of ascorbic acid results principally from the so-called “Smirnoff-Wheeler” pathway [24, 26]. The evolution of the level of ascorbic acid is linked to its involvement in cell growth, in plant defense against biotic and abiotic stress, and a strong presence in mitochondria, chloroplasts, nuclei, cytosol, peroxisomes and vacuoles [47, 49] following the slow-down in the growth rate and the photosynthetic capacities of grape berries during their development. The second phase of accumulation, corresponding to veraison, was marked for both acids by a drop in levels. This key phase in the evolution of grape berry metabolism is characterized, among other functions, by the reorientation of flows and physicochemical changes in cell walls [26, 50–53]. During this phase, the role played by ascorbic acid in grape-berry metabolism can be modified and redirected towards other physiological regulations [47], which would explain the stagnation of the content levels. This second storage phase was followed by a period of stabilization in 2013 and a slight increase in 2011 corresponding to berry maturation. During this phase two alternative processes of ascorbic-acid production may be put forward to explain the slight further accumulation of ascorbic acid in the 2011 vintage. It may be recycled from its oxidized form (L-dehydroascorbate, or DHA) under the action of a dependent glutathione (GSH)-DHA reductase [47]. Indeed, at the beginning of ripening, and proportional to sugar accumulation, the GSH form of glutathione accumulates in the berry [54, 55], potentially activating GSH-dependent DHA reductase enzymes which are present in the berry tissues [56]. An alternative process may be the recycling of ascorbate from carbons resulting from cell-wall degradation related to maturation phenomena [26, 51, 57, 58] and releasing D-galacturonate, a precursor of ascorbate under the consecutive action of D-galacturonic acid reductase, aldono-lactonase, and L-galactono 1,4lactone dehydrogenase [59]. A third hypothesis might be put forward that is in relation to the climatic conditions prevalent in August 2011 in the Charente vineyard (http://www.cognac.fr/cognac/_fr/4_pro/index.aspx?page=actualite&id=2809 and http://www.cognac.fr/cognac/_fr/4_pro/index.aspx?page=actualite&id=4018); following a period of severe drought, ripening took place under rainy conditions, which resulted in a phenomenon of exceptional vigor in the vines. The further accumulation of ascorbic acid in the berries may have been imported from the new young leaves, as is the case in tomatoes [60]. Furthermore, as was reported by Melino et al. [9], the concentrations measured were found to be very low, which would seem to confirm the hypothesis of the authors concerning the direct use of ascorbic acid, without storage in easily extractible cellular compartments.

### Tartrate and ascorbate: two different steps of the pathway

Gene-expression and protein-abundance studies, supplemented by correlation studies seem to indicate that the enzymes that are involved in the synthesis of tartaric acid do not belong to the enzyme suite at work in the Smirnoff-Wheeler pathway, and that the synthesis of tartaric acid is regulated in a different way from that of ascorbic acid.

All results confirm the hypothesis of the presence, in the young green berry, of five enzymes which are more or less dependent on each other [9, 34, 35]. These enzymes, comprising 5 genes (*VvGME, VvVTC2, VvVTC4, VvGLDH, VvL-GalDH)*, are involved in the synthesis of ascorbic acid. In a second step, the only enzyme known to date corresponding to the *VvL-IdnDH* gene is involved in the synthesis of tartrate but is perhaps not alone and/or is not involved only in tartrate synthesis. Furthermore, an apparent concordance was found between the genetic and protein profiles of the 5 enzymes of the Smirnoff-Wheeler pathway and the evolution of the ascorbic acid content of berries, especially during the herbaceous growth phase in 2011, suggesting a vintage effect. This effect would lead to a stimulation of the synthesis of ascorbate related to hot and dry conditions during this period. The relationship between genetic and protein profiles of Smirnoff-Wheeler enzymes and ascorbic-acid content may be the consequence of a vintage effect, particularly differences in exposure to sunlight at the beginning of the growth phase; 2011 was an early vintage with a lot of sunshine during the herbaceous growth period, whereas 2013 was a later vintage with lower than average sunshine during the herbaceous growth period (Fig. 2). Furthermore, some of these enzymes, such as *VvGLDH,* have light-dependent genes [10, 39].

*VvL-IdnDH* was found to differentiate itself from the previous genes ones as its profile of gene-expression and protein-abundance suggests that it is not part of the Smirnoff-Wheeler pathway, but that its regulation may be affected by ascorbate as a final product. Overall, the genes belonging to the Smirnoff-Wheeler pathway express themselves prior to *VvL-IdnDH,* whose maximum is observed after the peak of the genes of the Smirnoff-Wheeler pathway. The synthesis of tartaric acid seems to follow that of ascorbic acid, after activation of the synthesis of ascorbate. The results of the present study clearly highlight this phenomenon, which occurs between the first growth phase and veraison (VB), but it could occur much more prematurely, prior to flowering, as suggested by Melino et al. [9].

### L-IdnDH and tartrate biosynthesis

Very little is known about a hypothetical link between L-IdnDH enzyme activity and tartaric acid content. Observations made during the present study confirmed the biosynthesis of this acid via L-IdnDH, which is also consistent with the work of Melino et al. [9] who had demonstrated by genetic study that the maximum level of expression of genes coding for this enzyme was found at the beginning of berry growth. In the present study peaks of L-IdnDH enzyme activity and tartrate-content accumulation were always preceded by a period of intense *VvL-IdnDH* expression and L-IdnDH abundance, this being true for both the 2011 and 2013 vintages. An interesting observation concerns the difference in the levels of these parameters between 2011 and 2013. Whereas the level of *VvL-IdnDH* was higher during 2011, levels of L-IdnDH protein-abundance and enzyme activity were lower and tartrate content was higher (and vice versa during 2013). This finding suggests that when the climatic conditions are unfavorable, the L-IdnDH protein could be regulated prior to or during translation. This regulation could be linked with a cultivar effect, and it would be a “salvage” regulation allowing the enzyme to perform its function. The question arises as to whether this regulation is dependant on another pathway, or another factor such as GME which is apparently correlated with the L-IdnDH protein, or more probably an unknown regulator under tartrate feedback. L-IdnDH would thus not seem to be a key enzyme of the tartrate pathway [61]. Indeed, it is possible that this enzyme functions to a greater or lesser extent in coordination with other as yet unidentified enzymes, depending on climatic conditions.

Deluc et al. [43] for the Cabernet sauvignon cultivar and Wen et al. [41] for native European cultivars had already pointed out the parallel in the evolution of tartrate concentrations in the berry and the expression of *VvL-IdnDH* transcripts. The sudden increase in enzyme activity during the formation of the young berry is, therefore, closely correlated with *VvL-IdnDH* expression and protein translation of L-IdnDH. This first period of enzyme activity appears to be more a function of the developmental stage of the berry than dependent on environmental parameters, which is in line with the findings of Melino et al. [9] concerning the in-situ ascorbate (tartaric precursor) biosynthesis capacity of immature berries. However, the possible presence of a second peak of *VvL-IdnDH* expression in the course of ripening (VB) (observed during 2011 maturation), which may translate a vintage effect or a possible vine variety effect, has only been reported by Deluc et al. [43]. The resurgence of enzyme activity during maturation of the 2011 vintage has not been previously reported. It is also possible to link the present results to the work of Wen et al. [41], who demonstrated by immuno-localization the presence of L-IdnDH protein in the berry vacuoles and cytoplasm. This resumption of activity may be due to a vintage effect since, due to wet weather conditions in August 2011, there was renewed vegetative growth during ripening, which may have generated a new ascorbic acid pool that could have migrated to the berries [9] thus reactivating the enzyme. This hypothesis is in accordance with the evolution profile of ascorbic-acid content for which an increase was observed at the end of maturation. The resumption of activity may also be the result of the maturation of the cell walls, which, on breaking down, may release the enzyme proteins entrapped within them. In effect, Wen et al. [41] observed, by immunostaining, the presence of L-IdnDH in the cell-walls during the growth phase of the berries. During berry ripening, the cell-walls are broken down by pectolytic activity (PG, PME, XET) and release wall material into the cytoplasm [51–53]. In this way a renewed quantity of L-IdnDH protein may be created in this cell compartment and reactivated in the presence of ascorbic acid. The level of tartrate synthesis appears to be linked more to the level of *VvL-IdnDH* transcription than translation or the level of enzyme activity, which seems to be regulated by environmental factors during the maturation phase.

### Tartrate pathway and environmental effects

The comparative analysis of patterns of change in ascorbic-acid and tartaric-acid levels and in the activity of L-IdnDH showed that the first peak of enzyme activity occurred when ascorbate levels were highest, and was always followed by an accumulation of tartaric acid. This indicates a strong link between enzyme activity and the final product. In the same way, in 2011 the second accumulation phase of tartaric acid during maturation is in accordance with the evolution profile of enzyme activity, for which an increase was observed at the end of maturation. Moreover, in 2013 ascorbate and tartrate contents were lower than in 2011, the sun not being so strong during spring as in 2011. Levels of ascorbate and tartrate synthesis appear to be linked to the intensity of the sun [10, 49] and perhaps to higher degrees of water constraint during the spring [22]. However, tartaric acid synthesis in green berries was positively and strongly linked to the intensity of sunlight [8, 10, 62] and to water stress [22]. Thereby, strong sun during the start-of-growth phase and/or water constraint can boost ascorbate synthesis and thus tartrate synthesis as if tartrate were a form of storing a surplus of ascorbate.

Furthermore, between 2011 and 2013, it is interesting to note that the level of enzyme activity during the herbaceous growth phase was seen to be inversely proportional to the tartrate and ascorbate content. Thus, enzyme activity does not seem to be positively correlated with ascorbate availability; the tartaric content produced by a high level of ascorbate content will not induce a high level of L-IdnDH enzyme, and a high enzyme activity level will not produce a high level of tartrate content. This observation appears to confirm the hypothesis that either L-IdnDH is not the key enzyme of the tartrate pathway [61] or that it is not the only key enzyme in this pathway.

## Conclusions

The results of the present study highlighted that, as for other cultivars, Ugni blanc grape berries have two groups of expression profiles for the genes involved in the biosynthesis pathway of tartaric acid: those upstream of ascorbic acid, belonging to the Smirnoff-Wheeler pathway [24, 25, 47], partially cultivar- and environment-dependent, and those downstream of ascorbic acid, which are involved in the synthesis of tartaric acid (*VvL-IdnDH*) and which are modulated by a vintage effect. Moreover, a possible post-transcriptional “salvage” regulation was highlighted in this part of the tartaric acid pathway. This regulation depends on the climate, and probably permits the enzyme to perform its function despite unfavorable conditions.

In order to further understanding and knowledge of this pathway, different approaches may be considered. On the one hand, an exploratory genetic study of promoter structures would lead to a better understanding of the fine regulation of these genes. On the other hand, a comprehensive protein study would allow the identification of the intermediate protein between 5, keto-D-gluconic acid and L-tartaric acid, which potentially regulates the “salvage” pathway, potentially under tartrate feedback. Moreover, in the context of climate change, further study taking into account the vintage effect is required to complete these initial results in order to better anticipate the future physiological consequences for grapevines. A study of the earliest stages of this pathway is required in order to improve understanding of its process and the parameters that influence it.

## Methods

The field studies have been conducted in accordance with local legislation.

### Plant material

Grapes (*Vitis vinifera* L. cv Ugni blanc) were collected from a Cognac vineyard (Charente county, France) during the 2011 and 2013 growing seasons. Grape clusters were taken at six development stages, corresponding to the phenological stages defined by Eichhorn, K.W. and Lorenz, D.H. [63]: beginning of flowering (stage 19: FB), pea-sized (stage 31: PS), bunch closure (stage 33: BC), 10 % ripe berries (stage 35: VB), 80 % ripe berries (stage37: VE) and maturity (stage 38: H). 2011 was the earliest vintage in ten years in the Cognac region (Fig. 2a), thus the above development stages corresponded respectively to 0, 10, 31, 61, 81 and 103 days after anthesis (DAA). In comparison 2013 was the latest vintage for ten years in the Cognac region (Fig. 2b), thus the above development stages corresponded respectively to 0, 22, 43, 64, 78 and 106 DAA. For each stage, random samples of ten grape clusters were selected from ten vines, three times over, and immediately frozen in liquid nitrogen then stored at −80 °C prior to use.

### Physiological parameters and berry composition

Exactly 20 whole berries were randomly picked for each development stage and weighed to determine average berry weight (Table 1). In order to determine total acidity (Table 2), 20 grapes were randomly collected at regular intervals from the beginning of veraison until the harvest then pressed, the must being analyzed.

### Chemicals

Analytical grade or ultra-pure reagents were used for all experiments. All chemicals and reagents were obtained from Sigma-Aldrich, Fluka, or Merck. All biomolecular reagents were obtained from Promega.

### Total RNA isolation and RT-PCR

Berries were ground to a fine powder using a Mixer Mill MM400 (Retsch) under liquid nitrogen. Total RNA was isolated from 1 g of ground tissue, as described by Reid et al. [64]. Total concentration and purification were estimated by measuring absorbance at 260 nm on a microplate reader (BioTech, Colmar - France) using KC4-V3 software. The integrity of the extracted RNA was evaluated on 1.2 % agarose gel electrophoresis. No DNA contamination was detected by PCR amplification (40 cycles), and DNase-treated total RNA (2 μg) was reverse transcribed with oligo (dT)_15_, using MMLV reverse transcriptase (Promega, Charbonnières, France) according to the supplier’s instructions. cDNA syntheses for all RNA samples were performed simultaneously.

### Quantitative real-time PCR (qRT-PCR) analysis of gene transcription

*VvGME, VvVTC2, VvL-GalDH, VvGLDH, VvL-IdnDH* (transcript of interest) and *VvEFαI, VvUbiquitin* (reference genes) transcript levels were measured by real-time qRT-PCR, using the IQ-SYBR green supermix on a CFX96 ™ Real-Time PCR Detection System (Bio-Rad, Marnes La Coquette, France) monitored via Bio-Rad CFX manager 3.0 software. The reaction mixture (20 μL) contained 5 μL cDNA template and 0.25 μM of the forward and reverse primers specific to each gene (Additional file 3: Table S1). The thermal cycling conditions and primers for all genes studied are shown in Additional file 3: Table S1. Amplification specificity was verified for each gene product at the end of each run with melt curves. To verify the specificity of the primers, the amplification products were loaded onto a gel in order to verify the presence of a single band, and they were also sequenced at Eurofins MWG Operon (Ebersberg, Germany). PCR efficiency and standard curve linearity were also evaluated in preliminary experiments to ensure efficiency between 85 % and 115 % and an r^2^ value ≥ 0.95. The transcript mean levels for each gene were normalized to *VvEFαI* and *VvUbiquitin* with the 2^-ΔΔCt^ method, and to the “Flowering Beginning” stage. Three technical replicates were evaluated for each of the three biological replicates**.**

### Total protein extraction for enzyme and proteome studies

Protein extraction was performed according to the method described by Giribaldie et al. [65] and Gagné et al. [66], after making adaptations to samples: 100 mg of powdered plant material were homogenized in a 1.5 ml lysis buffer (Tris–HCl 0.5 M, pH8; Sucrose 1 %; DTT 100 mM; CaCl_2_ 25 mM; PVPP 1 %; TritonX100 0.1 %; Antiprotease 0.5 mL.L^−1^); the mixture was stirred at 4 °C for 2 h and centrifuged at 20000 g for 20 min (4 °C). The supernatant, or protein extract, was used to determine enzyme assay and proteome expression levels.

### Protein abundance level

The total protein concentration of the supernatant was determined by the Coomassie blue test [67] using the Coo Protein assay Kit (Interchim). The corresponding volume of protein concentration (10 μg/ml) was pipetted, homogenized with 1.5 ml of acetone/Trichloro-acetic acid (1/4; v/v), stored for 2 h, at −20 °C, and centrifuged at 20000 g for 20 min (4 °C). The resulting pellets were washed with acetone and centrifuged at 20000 g for 20 min (4 °C) twice over before being dried. The pellets were resuspended in a Laemmli buffer and the equivalent of 10 μg of protein was boiled for 2 min (90 °C) and loaded onto a 10 % SDS-PAGE. Migration was stopped once proteins had entered the separating gel. After Colloïdal Blue staining, a sole band was cut. As described elsewhere in the literature [68], proteins were digested, either directly (first dataset) or after reduction/alkylation (second dataset), by trypsin and the resulting peptide mixture was analyzed by nano LC MS/MS (Nano Liquid Chromatography Mass spectrometry) on an Ultimate 3000 nanoLC system (Dionex, The Netherlands) coupled either to a nanospray LTQ-Orbitrap XL (ThermoScientific, Bremen, Germany) or a nanospray Q-Exactive (ThermoScientific, Bremen) mass spectrometer. Ten microliters of peptide digests were loaded onto a 300 μm-inner-diameter × 5 mm C18 PepMapTM trap column (LC Packings) at a flow rate of 30 μL.min^−1^. The peptides were eluted from the trap column into an analytical 75 mm id × 15 cm C18 Pep-Map column (Dionex, The Netherlands; solvent A = 0.1 % formic acid in 5 % ACN; solvent B = 0.1 % formic acid in 80 % ACN)) with a 4–40 % linear gradient of solvent B in 105 min. The separation flow rate was set at 300 nL.min^−1^. The mass spectrometer operated in positive ion mode at 1.8 kV. On the Orbitrap, data were acquired in a data-dependent mode alternating an FTMS scan survey over the range m/z 300–1700 and six ion trap MS/MS scans with Collision Induced Dissociation (CID) as activation mode. MS/MS spectra were acquired using a 3 m/z unit ion isolation window and normalized collision energy of 35.

On the Q-Exactive, data were acquired in a data-dependent mode alternating a scan survey over the range m/z 300–2000 and 15 MS/MS scans with Higher-energy C-trap Dissociation (HCD) as activation mode. MS/MS spectra were acquired using a 3 m/z unit ion isolation window and normalized collision energy of 25. In both cases, only +2 and +3 charge states were selected for fragmentation. Dynamic exclusion duration was set to 30s. Data were searched by SEQUEST through Proteome Discoverer 1.4 (Thermo Fisher Scientific Inc.) against the UniProt *Vitis vinifera* L. Reference Proteome Set database (version 2014.07; 29,837 entries). Spectra from peptides higher than 5000 Da or lower than 350 Da were rejected. Datasets were searched with the following parameters: mass accuracy of the mono-isotopic peptide precursor and peptide fragments was set to 10 ppm and 0.8 Da respectively for the 2011 dataset (Orbitrap), or to 10 ppm and 0.02 Da respectively for the 2013 dataset (Q-Exactive). Only b- and y-ions were considered for mass calculation. Variable modifications were associated to oxidation of methionines (+16 Da) for the 2011 dataset, or to oxidation of methionines (+16 Da), asparagine or glutamine deamidation (+1 Da), cysteine propionamide (+71 Da) or carbamidomethylation (+57 Da) of cysteines for the 2013 dataset. Two missed trypsin cleavages were allowed. Peptide validation was performed using the Percolator algorithm [69] and only “high confidence” peptides were retained corresponding to a 1 % False Positive Rate at peptide level. Quantitative analysis was performed using the Progenesis LC-MS program. Features were detected and aligned across the samples. Volumes were integrated for 2–6 charge-state ions. Normalization on ratio median was performed. Protein abundance was calculated by summing the volume of corresponding peptides. Four proteins could be relatively quantified: GME, VTC4, L-GalDH, L-IdnDH (Additional file 4: Table S2). L-IdnDH, detection and analysis concerned the two isoforms from class II [44].

### L-IdnDH activity assay

L-IdnDH activity was measured by means of a spectrophotometer (Thermo spectronic-Genesys), following the change in NADH absorbance at 340 nm, at 30 °C, according to the method described by DeBolt et al. [8], with some modifications. The protein extract (15 μl) was pre-equilibrated in 100 mM Tris HCl (pH8)/330 μM NADH in a glass cuvette zeroed at A_340_nm prior to the addition of substrate (5-keto-D-gluconic acid) to a final concentration of 50 mM. The modification in absorbance was assayed after 4 min.

### Organic acid extraction and assay

Ascorbic acid and tartaric acid were extracted and analyzed by HPLC as described by Melino et al. [70].

### Statistical data treatment

All analyses were performed three times over. Two-way analysis of variance (ANOVA) could not be performed (non-normality of the residues and/or non-homogeneity of the variance). Non-parametric tests were, therefore, carried out. The statistical significance of differences was determined by the Freidman test (α = 0.05) for paired samples. Experimental data detected as being significantly different are indicated in the tables by an asterisk. Analysis by Spearman correlation matrices (Additional file 1: Figure S1, Additional file 2: Figure S2) (circle = *ρ* < 0.05) were performed using R software (packs: RColorBrewer; psych; corrplot). Color intensity and size of circles are proportional to the correlation coefficient (cf.: scale on the right).

## Abbreviations

BC, bunch closure (berries touching); FB, flowering beginning; GLDH, L-galactono-1, 4-lactone dehydrogenase; GME, GDP-D-mannose-3, 5 epimerase; H, harvest (maturity); L-GalDH, L-Galactose dehydrogenase; L-IdnDH, L-Idonate dehydrogenase; PS, pea-sized; VB, veraison beginning (10 % ripe berries); VE, verasion end (80 % ripe berries); VTC2, GDP-L-galactose phosphorylase; VTC4, L-galactose-1-phosphate dehydrogenase; *VvGLDH*, *Vitis vinifera* L. L-GLDH gene; *VvGME*, *Vitis vinifera* L. GME gene; *VvL-GalDH*, *Vitis vinifera* L. L-GalDH gene; *VvL-IdnDH*, *Vitis vinifera* L. L-IdnDH gene; *VvVTC2*, *Vitis vinifera* L. VTC2 gene; *VvVTC4*, *Vitis vinifera* L. VTC4 gene.

## Additional files

Additional file 1: Figure S1. Comparison of the Spearman correlation matrices; vintage 2011 (circle = *ρ* < 0.05; color intensity and size of circles are proportional to the correlation coefficient) obtained with the statistical analysis of all data: growth phase data (**A**) and maturation phase data (**B**). (DOCX 1344 kb) Additional file 2: Figure S2. Comparison of the Spearman correlation matrices; vintage 2013 (circle = *ρ* < 0.05; color intensity and size of circles are proportional to the correlation coefficient) obtained with the statistical analysis of all data: growth phase data (**A**) and maturation phase data (**B**). (DOCX 2835 kb) Additional file 3: Table S1. Primers and thermal cycling conditions for *VvGME*, *VvVTC2, VvL-GalDH, VvGLDH, VvL-IdnDH, VvEFαI, VvUbiquitine* used to Q-PCR study. (XLSX 12 kb) Additional file 4: Table S2. Compilation of proteomic data concerning the four proteins of interest. (ZIP 824 kb)

## References

[1] Ribéreau-Gayon G (1968). Etude des mecanismes de synthese et de transformation de l’acide malique, de l’acide tartrique et de l’acide citrique chez vitis vinifera L. Phytochemistry.

[2] Ruffner HP, Hawker JS, Hale CR (1976). Temperature and enzymic control of malate metabolism in berries of Vitis vinifera. Phytochemistry.

[3] Iland PG, Coombe BG (1988). Malate, Tartrate, Potassium, and Sodium in Flesh and Skin of Shiraz Grapes During Ripening: Concentration and Compartmentation. Am J Enol Vitic.

[4] Terrier N, Sauvage FX, Ageorges A, Romieu C (2001). Changes in acidity and in proton transport at the tonoplast of grape berries during development. Planta.

[5] Martinez-Esteso MJ, Selles-Marchart S, Lijavetzky D, Pedreno MA, Bru-Martinez R (2011). A DIGE-based quantitative proteomic analysis of grape berry flesh development and ripening reveals key events in sugar and organic acid metabolism. J Exp Bot.

[6] Saito K, Kasai Z (1982). Conversion of L-ascorbic acid to L-idonic acid, L-idono-γ-lactone ane 2-keto-L-idonic acid in slices of immature grapes. Plant Cell Physiol.

[7] Malipiero U, Ruffner HP, Rast DM (1987). Ascorbic to tartaric acid conversion in grapevines. J Plant Physiol.

[8] DeBolt S, Cook DR, Ford CM (2006). L-tartaric acid synthesis from vitamin C in higher plants. Proc Natl Acad Sci U S A.

[9] Melino V, Soole K, Ford C (2009). Ascorbate metabolism and the developmental demand for tartaric and oxalic acids in ripening grape berries. BMC Plant Biol.

[10] Melino VJ, Hayes MA, Soole KL, Ford CM (2011). The role of light in the regulation of ascorbate metabolism during berry development in the cultivated grapevine Vitis vinifera L. J Sci Food Agric.

[11] Loewus FA (1999). Biosynthesis and metabolism of ascorbic acid in plants and of analogs of ascorbic acid in fungi. Phytochemistry.

[12] DeBolt S, Hardie J, Tyerman S, Ford CM (2004). Composition and synthesis of raphide crystals and druse crystals in berries of Vitis vinifera L. cv. Cabernet Sauvignon: Ascorbic acid as precursor for both oxalic and tartaric acids as revealed by radiolabelling studies. Aust. J. Grape Wine Res.

[13] Saito K, Kasai Z (1984). Synthesis of L-(+)-tartaric acid from L-ascorbic acid via 5-keto-D-gluconic acid in grapes. Plant Physiol.

[14] Ruffner HP (1982). Metabolism of tartaric and malic acids in Vitis: a review. Vitis.

[15] Boitaud L, Dumot V, Ferrari G, Lurton L (2010). Constat et incidence du changement climatique dans la région de Cognac.

[16] Neethling E, Barbeau G, Bonnefoy C, Quénol H (2012). Change in climate and berry composition for grapevine varieties cultivated in the Loire Valley. Clim Res.

[17] Sadras VO, Petrie PR, Moran MA (2013). Effects of elevated temperature in grapevine. II juice pH, titratable acidity and wine sensory attributes. Aust J Grape Wine Res.

[18] Mira de Orduña R (2010). Climate change associated effects on grape and wine quality and production. Food Res Int.

[19] Hannah L, Roehrdanz PR, Ikegami M, Shepard AV, Shaw MR, Tabor G (2013). Climate change, wine, and conservation. Proc Natl Acad Sci.

[20] Ishikawa T, Dowdle J, Smirnoff N (2006). Progress in manipulating ascorbic acid biosynthesis and accumulation in plants. Physiol Plant.

[21] Ford CM (2012). The biochemistry of organic acids in the grape. Biochem Grape Berr.

[22] Gallie DR (2013). Increasing Vitamin C Content in Plant Foods to Improve Their Nutritional Value—Successes and Challenges. Nutrients.

[23] Zechmann B (2014). Compartment-specific importance of glutathione during abiotic and biotic stress. Plant Physiol.

[24] Wheeler GL, Jones MA, Smirnoff N (1998). The biosynthetic pathway of vitamin C in higher plants. Nature.

[25] Smirnoff N (2001). l-Ascorbic acid biosynthesis. Vitam Horm.

[26] Cruz-Rus E, Botella MA, Valpuesta V, Gomez-Jimenez MC (2010). Analysis of genes involved in l-ascorbic acid biosynthesis during growth and ripening of grape berries. J Plant Physiol.

[27] Linster CL, Clarke SG (2008). l-Ascorbate biosynthesis in higher plants: the role of VTC2. Trends Plant Sci.

[28] Gilbert L, Alhagdow M, Nunes-Nesi A, Quemener B, Guillon F, Bouchet B (2009). GDP-d-mannose 3,5-epimerase (GME) plays a key role at the intersection of ascorbate and non-cellulosic cell-wall biosynthesis in tomato. Plant J.

[29] Wolucka BA, Van Montagu M (2007). The VTC2 cycle and the de novo biosynthesis pathways for vitamin C in plants: An opinion. Phytochemistry.

[30] Voxeur A, Gilbert L, Rihouey C, Driouich A, Rothan C, Baldet P (2011). Silencing of the GDP-d-mannose 3,5-Epimerase Affects the Structure and Cross-linking of the Pectic Polysaccharide Rhamnogalacturonan II and Plant Growth in Tomato. J Biol Chem.

[31] Laing WA, Wright MA, Cooney J, Bulley SM (2007). The missing step of the L-galactose pathway of ascorbate biosynthesis in plants, an L-galactose guanyltransferase, increases leaf ascorbate content. Proc Natl Acad Sci U S A.

[32] Linster CL, Gomez TA, Christensen KC, Adler LN, Young BD, Brenner C (2007). Arabidopsis VTC2 encodes a GDP-L-galactose phosphorylase, the last unknown enzyme in the smirnoff-wheeler pathway to ascorbic acid in plants. J Biol Chem.

[33] Conklin PL, Gatzek S, Wheeler GL, Dowdle J, Raymond MJ, Rolinski S (2006). Arabidopsis thaliana VTC4 encodes L-galactose-1-P phosphatase, a plant ascorbic acid biosynthetic enzyme. J Biol Chem.

[34] Ioannidi E, Kalamaki MS, Engineer C, Pateraki I, Alexandrou D, Mellidou I (2009). Expression profiling of ascorbic acid-related genes during tomato fruit development and ripening and in response to stress conditions. J Exp Bot.

[35] Li M, Ma F, Guo C, Liu J (2010). Ascorbic acid formation and profiling of genes expressed in its synthesis and recycling in apple leaves of different ages. Plant Physiol Biochem.

[36] Gatzek S, Wheeler GL, Smirnoff N (2002). Antisense suppression of L-galactose dehydrogenase in Arabidopsis thaliana provides evidence for its role in ascorbate synthesis and reveals light modulated L-galactose synthesis. Plant J.

[37] Do Nascimento JRO, Higuchi BK, Gómez MLPA, Oshiro RA, Lajolo FM (2005). l-Ascorbate biosynthesis in strawberries: l-Galactono-1,4-lactone dehydrogenase expression during fruit development and ripening. Postharvest Biol Technol.

[38] Alhagdow M, Mounet F, Gilbert L, Nunes-Nesi A, Garcia V, Just D (2007). Silencing of the Mitochondrial Ascorbate Synthesizing Enzyme l-Galactono-1,4-Lactone Dehydrogenase Affects Plant and Fruit Development in Tomato. Plant Physiol.

[39] Bartoli CG, Yu J, Gómez F, Fernández L, McIntosh L, Foyer CH (2006). Inter-relationships between light and respiration in the control of ascorbic acid synthesis and accumulation in Arabidopsis thaliana leaves. J Exp Bot.

[40] Dowdle J, Ishikawa T, Gatzek S, Rolinski S, Smirnoff N (2007). Two genes in Arabidopsis thaliana encoding GDP-L-galactose phosphorylase are required for ascorbate biosynthesis and seedling viability. Plant J.

[41] Wen YQ, Li J-M, Zhang ZZ, Zhang YF, Pan QH (2010). Antibody preparation, gene expression and subcellular localization of L-idonate dehydrogenase in grape berry. Biosci Biotechnol Biochem.

[42] Wen YQ, Cui J, Zhang Y, Duan Q, Pan QH (2014). Comparison of organic acid levels and L-IdnDH expression in Chinese-type and European-type grapes. Euphytica.

[43] Deluc LG, Grimplet J, Wheatley MD, Tillett RL, Quilici DR, Osborne C (2007). Transcriptomic and metabolite analyses of Cabernet Sauvignon grape berry development. BMC Genomics.

[44] Jia Y, Wong DCJ, Sweetman C, Bruning JB, Ford CM. New insights into the evolutionary history of plant sorbitol dehydrogenase. BMC Plant Biol. 2015;15. 10.1186/s12870-015-0478-5 PMC4404067 25879735

[45] Ojeda H, Deloire A, Carbonneau A (2001). Influence of water deficits on grape berry growth. Vitis.

[46] Ojeda H, Andary C, Kraeva E, Carbonneau A, Deloire A (2002). Influence of pre- and postveraison water deficit on synthesis and concentration of skin phenolic compounds during berry growth of Vitis vinifera cv. Shiraz. Am J Enol Vitic.

[47] Smirnoff N, Wheeler GL (2000). Ascorbic acid in plants: Biosynthesis and function. Crit Rev Plant Sci.

[48] Ollat N, Diakou-Verdin P, Carde JP, Barrieu F, Gaudillere JP, Moing A (2002). Grape berry development : A review. J Int Sci Vigne Vin.

[49] Zechmann B (2011). Subcellular distribution of ascorbate in plants. Plant Signal Behav.

[50] Coombe BG, McCarthy MG (2000). Dynamics of grape berry growth and physiology of ripening. Aust J Grape Wine Res.

[51] Brummell DA, Cin VD, Crisosto CH, Labavitch JM (2004). Cell wall metabolism during maturation, ripening and senescence of peach fruit. J Exp Bot.

[52] Deytieux C, Geny L, Lapaillerie D, Claverol S, Bonneu M, Donèche B (2007). Proteome analysis of grape skins during ripening. J Exp Bot.

[53] Goulao LF, Oliveira CM (2008). Cell wall modifications during fruit ripening: when a fruit is not the fruit. Trends Food Sci Technol.

[54] Adams DO, Liyanage C (1993). Glutathione Increases in Grape Berries at the Onset of Ripening. Am J Enol Vitic.

[55] Okuda T, Yokotsuka K (1999). Levels of Glutathione and Activities of Related Enzymes During Ripening of Koshu and Cabernet Sauvignon Grapes and During Winemaking. Am J Enol Vitic.

[56] Foyer CH, Noctor G (2011). Ascorbate and Glutathione: The Heart of the Redox Hub. Plant Physiol.

[57] Fougère-Rifot M, Cholet C, Bouard J (1996). Evolution of the hypodermal cells of grape berry during their transformation in pulp cells. J Int Sci Vigne Vin.

[58] Huang XM, Huang HB, Wang HC (2005). Cell walls of loosening skin in post-veraison grape berries lose structural polysaccharides and calcium while accumulate structural proteins. Sci Hortic.

[59] Isherwood FA, Chen YT, Mapson LW (1954). Synthesis of L-ascorbic acid in plants and animals. Biochem J.

[60] Badejo AA, Wada K, Gao Y, Maruta T, Sawa Y, Shigeoka S (2012). Translocation and the alternative D-galacturonate pathway contribute to increasing the ascorbate level in ripening tomato fruits together with the D-mannose/L-galactose pathway. J Exp Bot.

[61] Shangguan L, Sun X, Zhang C, Mu Q, Leng X, Fang J (2015). Genome identification and analysis of genes encoding the key enzymes involved in organic acid biosynthesis pathway in apple, grape, and sweet orange. Sci Hortic.

[62] DeBolt S, Ristic R, Hand PG, Ford CM (2008). Altered light interception reduces grape berry weight and modulates organic acid biosynthesis during development. HortSci.

[63] Eichhorn KW, Lorenz DH (1977). Phänologische Entwicklungsstadien der Rebe.

[64] Reid KE, Olsson N, Schlosser J, Peng F, Lund ST. An optimized grapevine RNA isolation procedure and statistical determination of reference genes for real-time RT-PCR during berry development. BMC Plant Biol. 2006;6. 10.1186/1471-2229-6-27 PMC1654153 17105665

[65] Giribaldi M, Perugini I, Sauvage FX, Schubert A (2007). Analysis of protein changes during grape berry ripening by 2-DE and MALDI-TOF. PROTEOMICS.

[66] Gagné S, Lacampagne S, Claisse O, Gény L (2009). Leucoanthocyanidin reductase and anthocyanidin reductase gene expression and activity in flowers, young berries and skins of Vitis vinifera L. cv. Cabernet-Sauvignon during development. Plant Physiol Biochem.

[67] Bradford MM. A rapid and sensitive method for the quantitation of microgram quantities of protein utilizing the principle of protein-dye binding. Anal Biochem. 1976;72. 10.1016/0003-2697(76)90527-3 942051

[68] Lanotte P, Perivier M, Haguenoer E, Mereghetti L, Burucoa C, Claverol S (2013). Proteomic Biomarkers Associated with Streptococcus agalactiae Invasive Genogroups. PLoS ONE.

[69] Käll L, Canterbury JD, Weston J, Noble WS, MacCoss MJ (2007). Semi-supervised learning for peptide identification from shotgun proteomics datasets. Nat Methods.

[70] Melino VJ, Soole KL, Ford CM (2009). A method for determination of fruit-derived ascorbic, tartaric, oxalic and malic acids, and its application to the study of ascorbic acid catabolism in grapevines. Aust J Grape Wine Res.

