# Supplementary figures and images for: Tartaric acid pathways in Vitis vinifera L. (cv. Ugni blanc): a comparative study of two vintages with contrasted climatic conditions
Source: BMC Plant Biol. 2016 Jun 28;16:144. doi: 10.1186/s12870-016-0833-1 (PMC4924324; doi:10.1186/s12870-016-0833-1)

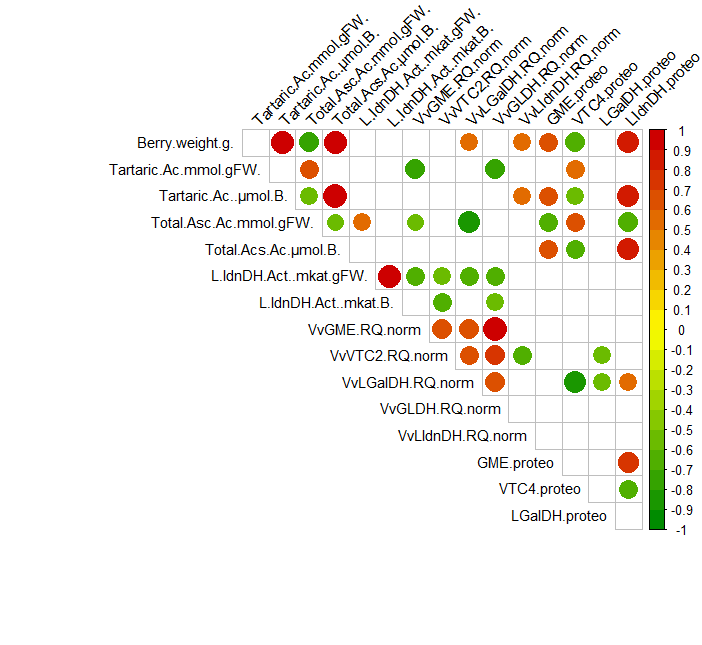


**A :** Dataset of 2011 vintage : growth phase


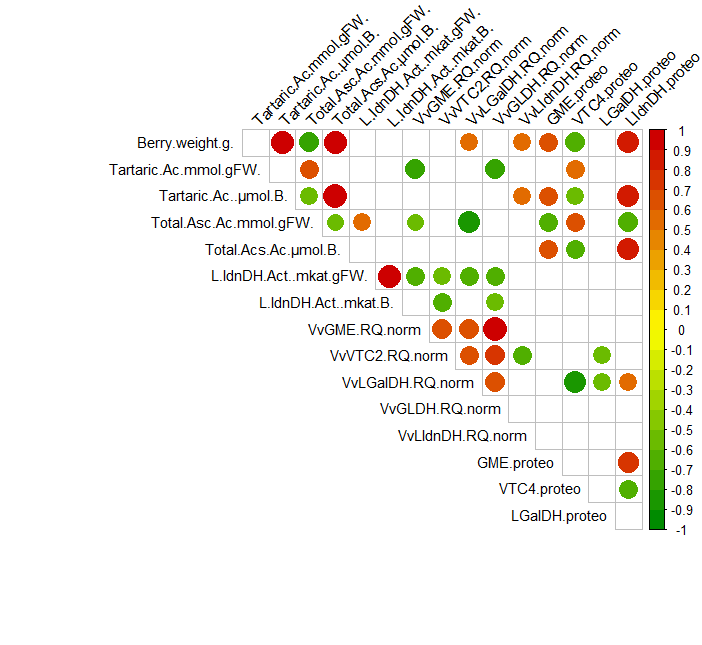


**B :** Dataset of 2011 vintage : maturation phase

**Figure S1**.

Supplement: Additional file 1: Figure S1. — Comparison of the Spearman correlation matrices; vintage 2011 (circle = ρ < 0.05; color intensity and size of circles are proportional to the correlation coefficient) obtained with the statistical analysis of all data: growth phase data (A) and maturation phase data (B). (DOCX 1344 kb) [file 12870_2016_833_MOESM1_ESM.docx]

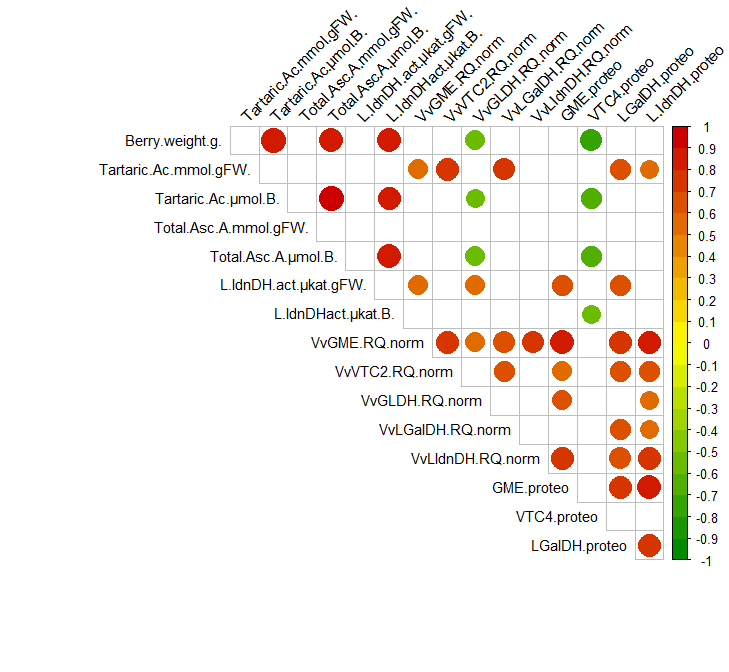


**A :** Dataset of 2013 vintage : growth phase


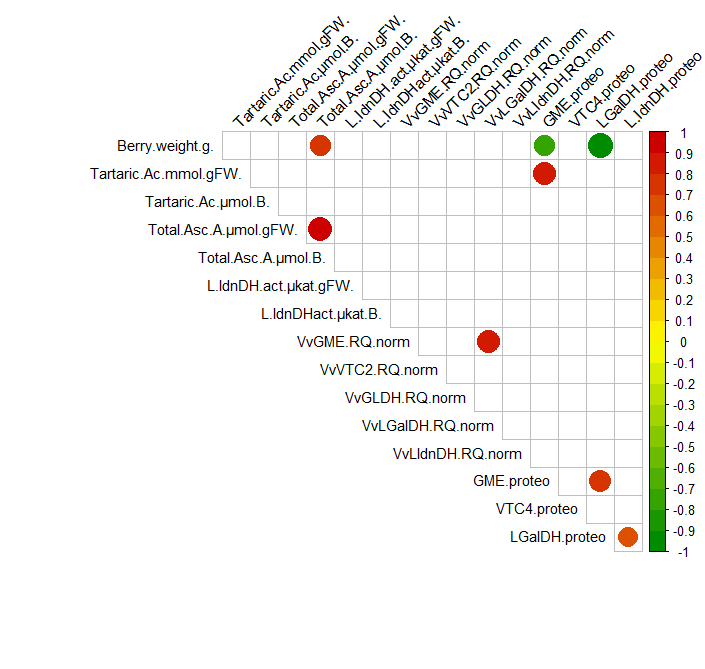


**B :** Dataset of 2013 vintage : maturation phase

**Figure S2**.

Supplement: Additional file 2: Figure S2. — Comparison of the Spearman correlation matrices; vintage 2013 (circle = ρ < 0.05; color intensity and size of circles are proportional to the correlation coefficient) obtained with the statistical analysis of all data: growth phase data (A) and maturation phase data (B). (DOCX 2835 kb) [file 12870_2016_833_MOESM2_ESM.docx]
